# Cancer Stem Cells in Renal Cell Carcinoma: Origins and Biomarkers

**DOI:** 10.3390/ijms241713179

**Published:** 2023-08-24

**Authors:** Francesco Lasorsa, Monica Rutigliano, Martina Milella, Matteo Ferro, Savio Domenico Pandolfo, Felice Crocetto, Riccardo Autorino, Michele Battaglia, Pasquale Ditonno, Giuseppe Lucarelli

**Affiliations:** 1 Department of Precision and Regenerative Medicine and Ionian Area-Urology, Andrology and Kidney Transplantation Unit, University of Bari “Aldo Moro”, 70124 Bari, Italy; 2 Division of Urology, European Institute of Oncology, IRCCS, 71013 Milan, Italy; 3 Department of Neurosciences and Reproductive Sciences and Odontostomatology, University of Naples “Federico II”, 80131 Naples, Italy; 4 Department of Urology, Rush University Medical Center, Chicago, IL 60612, USA

**Keywords:** renal cell carcinoma, cancer stem cell, CD133, markers, treatment

## Abstract

The term “cancer stem cell” (CSC) refers to a cancer cell with the following features: clonogenic ability, the expression of stem cell markers, differentiation into cells of different lineages, growth in nonadhesive spheroids, and the in vivo ability to generate serially transplantable tumors that reflect the heterogeneity of primary cancers (tumorigenicity). According to this model, CSCs may arise from normal stem cells, progenitor cells, and/or differentiated cells because of striking genetic/epigenetic mutations or from the fusion of tissue-specific stem cells with circulating bone marrow stem cells (BMSCs). CSCs use signaling pathways similar to those controlling cell fate during early embryogenesis (Notch, Wnt, Hedgehog, bone morphogenetic proteins (BMPs), fibroblast growth factors, leukemia inhibitory factor, and transforming growth factor-β). Recent studies identified a subpopulation of CD133+/CD24+ cells from ccRCC specimens that displayed self-renewal ability and clonogenic multipotency. The development of agents targeting CSC signaling-specific pathways and not only surface proteins may ultimately become of utmost importance for patients with RCC.

## 1. Introduction

Renal cell carcinoma accounts for 3–5% of all human cancers and, according to the American Cancer Society’s 2023 estimates, 81,800 new cases will be diagnosed in the USA, and 14,890 individuals will die from this disease [1]. The most prevalent histological subtypes of RCC are clear cell RCC (ccRCC), which can be considered as a metabolic disease due to the radical metabolic adaptations observed in cancer cells [2,3,4,5,6,7,8,9,10,11,12]. Surgery is the gold standard treatment of localized disease, although one-third of patients are diagnosed with metastatic diseases and/or will develop disease recurrence after surgery [13,14,15,16,17,18,19]. Ongoing research into the tumor microenvironment (TME) led to the approval of molecular target-based agents including tyrosine kinase inhibitors (TKIs) and immune checkpoint inhibitors (ICIs) [20,21,22,23]. Combinations of TKI and ICI are recommended as first-line therapy for advanced RCC even if patients may develop drug resistance over time [24]. In this scenario, cancer stem cells (CSCs) are thought to play a crucial role in recurrence and metastasis in RCC patients. Recent research has characterized CSCs in kidney cancer, evaluated their presence, and compared their molecular profile to that of their normal counterparts. In this review, we aim to describe the main features of CSCs and their possible role in RCC biology.

## 2. Cancer Stem Cells: Definition and Properties

**Figure 1:**
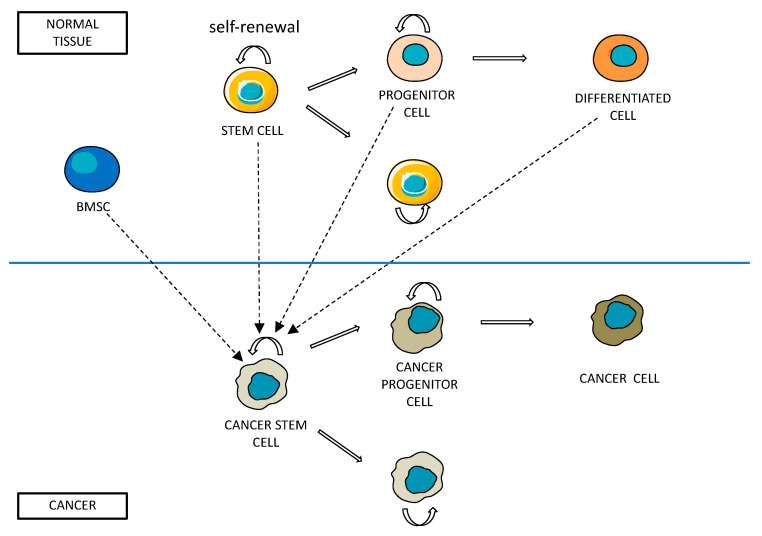
Summary of hypothesis leading to the origin of cancer stem cells. Self-renewal depends on asymmetric division of both normal and cancer stem cells. BMSC: bone marrow-derived stem cell.

Different models have been proposed over the years to describe tumor development, progression, and heterogeneity. According to the clonal (or stochastic) model, differentiated cells undergo multiple mutations over time. In line with Darwinian theory, cell populations carrying a mutation that confers proliferative and/or survival advantage will replace those cells that lack it. The term “cancer stem cell” (CSC) refers to a cancer cell with the following features: clonogenic ability, the expression of stem cell markers, growth in nonadhesive spheroids, and the ability to differentiate into cells of different lineages and to generate in vivo serially transplantable tumors that reflect the heterogeneity of primary cancers (tumorigenicity). In immune-compromised mice (i.e., nonobese diabetic (NOD)/severe combined immunodeficiency mice (SCID)), CSCs appear to be the only cells able to generate a new tumor. Self-renewal depends on asymmetric cell divisions, which give rise to a quiescent stem cell and a committed progenitor that will differentiate. During the differentiation of committed progenitor cells, the expression of genes required for self-renewal (i.e., Oct4, Nanog, and Sox2) is repressed. In contrast, lineage-specific genes are switched on. In 1994, Dick and co-authors first isolated CSCs from acute myeloid leukemia (AML) patients [25]. The cancer stem cell model or hierarchical model states that growth and propagation depend on CSCs, from which descendants will form a tumor (Figure 1). However, the majority of populations in the tumor mass behave as progenitor cells (or transit-amplifying cells) with limited proliferative potential. Progenitor cells may represent intermediates between stem cells and fully differentiated ones. According to this new model, CSCs may arise from normal stem cells, progenitor cells, and/or differentiated cells because of striking genetic/epigenetic mutations [26]. Another theory suggests that CSCs may be derived from the fusion of tissue-specific stem cells with circulating bone marrow stem cells (BMSCs). More recently, Kreso et al. [27] proposed a unifying model of clonal evolution applied to CSCs. CSCs may acquire further mutations and generate new stem branches. Tumor cells in the non-CSCs subpopulation may undergo the epithelial–mesenchymal transition (EMT) and acquire CSC-like features, thus enhancing tumor heterogeneity [27]. CSCs use signaling pathways similar to those controlling cell fate during early embryogenesis (Notch, Wnt, Hedgehog, bone morphogenetic proteins (BMPs), fibroblast growth factors, leukemia inhibitory factor, and transforming growth factor-β). While transiently activated in normal stem cells, they may encourage a long-lasting activation state in cancer stem cells [28]. CSCs are thought to be the main cause of recurrence and resistance to therapy and appear inherently resistant to chemo- and radiotherapy [29,30]. By promoting their active efflux, multidrug resistance (MDR) transporters (such as ATP-binding cassette—ABC) prevent drug accumulation in CSCs. ATP-binding cassette, sub-family B, member 5 (ABCB5) is a plasma membrane protein involved in the transport of small ions, sugars, peptides, and organic molecules (such as drugs) against a concentration gradient by ATP hydrolysis. It is overexpressed in CSCs of melanoma, liver, and colorectal cancers where it is thought to be associated with progression, chemotherapy resistance, and recurrence [31]. It is believed that the effect of inhibiting a single ABC transporter may be counteracted by the simultaneous expression of several MDR transporters. Active DNA repair mechanisms may also explain their resistance to conventional therapies. Radiotherapy results in the production of reactive oxygen species (ROS) in cancer cells. Enhanced free radical scavenging systems (i.e., N-acetylcysteine) appear to cause lower ROS levels in both human and mouse mammary CSCs compared to more differentiated tumor cells [32]. The intracellular levels of reduced glutathione (GSH) appear to be controlled by CD44, which interacts with a glutamate–cysteine transporter [33]. Ataxia-teleangectasia mutated (ATM) and ataxia-teleangectasia mutated RAD3 (ATR) protein kinases are key sensors of DNA damage and drive the activation of checkpoint kinase 1 (CHK1) and 2 (CHK2) leading to DNA repair. These may contribute to therapy resistance and their pharmacological inhibition sensitized CSCs to radiotherapy [34]. In stress conditions (hypoxia, ischemia, or nutrition deprivation), autophagic machinery may provide nutrients and energy [35,36]. Ovarian CSCs exhibited higher basal autophagy than non-CSCs so their inhibition might reduce chemosensitivity [37]. Hypoxia modulates gene expression mainly by promoting hypoxia-inducible factor-1α and 2α (HIF-1α, HIF-2α) or phosphatidiyl-inositol-3-kinase (PI3K/AKT). PI3K/AKT promotes HIF-1α/HIF-2α as a feedback loop. In pancreatic CSCs, the upregulation of VEGF, IL-6, Nanog, Oct4, and EZH2 support their invasion, migration, and angiogenesis [38,39]. Although the molecular mechanism is unclear, ferroptosis is a recently described form of cell death. Iron cycling (from oxidized to reduced forms) may produce free radicals responsible for lipid peroxidation and DNA damage within cells. CSCs are typically distinguished as having a greater intracellular iron content [40,41]. The growth of CSCs in ovarian cancer was reduced when their intracellular storage was reduced, indicating a connection between ferroptosis and CSCs [42]. Further research might provide deeper insights into the ferroptosis- and autophagy-mediated resistance of CSCs. In different solid and hematological malignancies, CSC signaling pathways may be associated with chemoresistance. In neuroblastoma, the Wnt/β-catenin axis supports MDR1 gene expression. Notch and Hedgehog may contribute to temozolomide resistance in glioma CD133+ CSCs and to platinum resistance in ovarian CSCs [43].

Chemotherapy and radiotherapy mainly target proliferative cells. Thus, as long as cytotoxic stimuli occur, cells may adopt a transient state of slow proliferation rate known as drug tolerance. This condition may be reverted after the cessation of the stimuli. In contrast, environmental factors may stabilize this quiescent condition into a short-, medium- or long-term dormancy. Dormant cells are typically arrested in G0 or the G0/G1 transition [44,45]. Evidence suggests that both extrinsic and intrinsic cues may induce cellular dormancy. The downregulation of the integrin receptor and downstream RAS-ERK/MAPK and PI3K-AKT signaling may drive cellular dormancy. Stress-induced pathways (i.e., the unfolded protein response) have also been implicated in cellular dormancy via p38/ERK [46]. CSCs are capable of alternating between periods of rapid growth and dormancy (CSC plasticity). The identification and targeting of CSCs are further complicated by their plasticity. The dormant phenotype has emerged to be crucial for metastasis and therapy resistance in certain malignancies. Indeed, non-dividing dormant CSCs become insensitive to conventional antiproliferative drugs [47]. Finally, long-term recurrence is caused by dormant tumor cells that have survived multiple therapeutic cycles. Microenvironmental cues or therapies may be responsible for cellular senescence in CSCs. It is a possibly reversable terminal cellular state because of growth arrest (cell cycle arrest in the G1 phase). Senescent cells are capable of secreting a series of cytokines (senescence-associated secretory phenotype (SASP)), which may support tumorigenesis and even stemness [48].

ICIs (anti-CTLA-4 and anti-PD-1 pathway) have been demonstrated to induce durable regression in a variety of tumors. Similar to conventional chemotherapy and radiotherapy, not every type of cancer or patient responds effectively to ICI, and CSCs may play a role in immunotherapy resistance. The immune privilege of CSCs sets them apart from differentiated tumor cells, but immunosuppressive pathways vary in a tissue- and cancer-dependent way [49]. CD47-mediated phagocytosis is prevented by the overexpression of SIRPα; the interaction of CD24 with its receptor Siglec-10 limits both T cells and macrophage activities. In CSCs, MTDH and SND1 interact as a stress response: this impairs mRNA encoding components of the antigen-presenting machinery [50]. T cell activity is further blocked by the increased expression of immune checkpoint molecules (PD-L1 and TIM3) upon PI3K/Akt/β-catenin axis activation. Bidirectional crosstalk occurs in the TME between CSCs and other cells. CSCs may activate cancer-associated fibroblasts (CAFs) to secrete hyaluronan and alteration in the extracellular matrix (ECM) may affect immune infiltration. The inhibition of Wnt, Notch, and Hedgehog has been approached to overcome CSCs’ immune privilege. Indeed, melanoma progression has been reduced by anti-CTL4 therapy combined with Wnt signaling inhibition [51].

## 3. Metabolism of Stem Cells and Cancer Cells

Glucose and glutamine are essential macromolecules for both pluripotent stem cells (PSCs) and cancer cells [52]. Not only do they represent sources for ATP and NAD(P)H production, but their catabolism also generates precursors for de novo lipid, protein, and nucleic acid biosynthesis. The Warburg effect is a hallmark of all rapidly proliferating mammalian cells. Despite high oxygen levels, glucose is oxidized to lactate. Glycolytic flux decreases during PSCs differentiation, but is restored during the reprogramming of differentiated cells to the pluripotent state [53,54]. It has been noted that transcription factors establishing pluripotency may directly regulate the glycolytic phenotype. Indeed, Oct4 binds the loci encoding for glycolysis enzymes, thereby promoting this pathway [55]. In addition, several metabolic intermediates enable chromatin modifications, which in turn regulate gene expression programs involved in self-renewal and lineage differentiation. Nevertheless, the metabolism of CSCs remains poorly understood since they exhibit features of both normal stem cells and cancer cells [56,57]. Contrasting results have been obtained when profiling CSCs metabolism in different cancer types. Interestingly, CSCs may undergo metabolic reprogramming in a context-dependent way (oxygen tension, pH, and glucose availability in TME) and in relation to genetic mutations and signaling pathways. Hence, CSC metabolism can switch from aerobic glycolysis to oxidative phosphorylation (OXPHOS) [58]. In response to hypoxia, glycolytic enzymes may be upregulated to switch to a more glycolytic phenotype, whereas CSCs rely mainly on OXPHOS in glucose-deprived conditions. Glutamine metabolism may supplement glucose by providing intermediates for nucleotides, amino acids, and lipids synthesis. Eventually, lipid metabolism is affected in CSCs. Higher amounts of lipid droplets and CD133 expression in CSCs have been associated with greater clonogenicity and tumor-forming capability [59].

## 4. Nephrogenesis and Signaling Pathways

Stem cells are known to be able to self-renew and differentiate into one or more types of mature cells. Adult stem cells are located in a specialized milieu known as the niche, and secreted effectors play crucial roles in controlling stem cell maintenance, proliferation, survival, activation and differentiation within the niche. Thus, surface receptors can be activated as well as intracellular signal cascades, which will ultimately modulate gene expression. Moreover, stem cell programming also depends on intercellular communication among stem cells, niche supporting cells, and their differentiated daughter cells [60]. Previous studies on invertebrates (Drosophila) and mammalians provided deep molecular insights in stem cell signaling pathways (Wnt, Notch, Hedgehog, Hippo, Jak/STAT, BMP, etc.). Some of these signaling pathways control self-renewal and proliferation while others are involved in progenitor cell differentiation. Human nephrogenesis consists of three embryonic stages: pronephros, mesonephros, and metanephros, which will eventually develop into kidneys. The ureteric bud from the nephric duct migrates to the metanephric mesenchyme and invades it. Glial-derived neurotrophic factor (GDNF) and hepatocyte growth factor (HGF) released from the metanephric mesenchyme promote ureteric bud branching into the urinary system [61]. Except for collecting duct epithelial cells, which come from the ureteric bud, nephron epithelial cells, myofibroblasts, and smooth muscle cells derive from the metanephric mesenchyme. While branching, the ureteric bud facilitates mesenchymal survival and differentiation by releasing a variety of factors such as Wnt proteins, fibroblast growth factors (FGFs), and leukemia inhibitory factor (LIF). The mesenchymal-to-epithelial transition (MET) is essential for the differentiation of mesenchymal cells (mesenchymal cap) into nephrons [62]. Cap mesenchyme cells (expressing Osr1, Pax2, Wt1, Six2, and Cited1) represent the main source of renal progenitor cells. Six2 expression decreases as long as cells undergo MET and it is absent in mature kidneys [63]. The Wnt9b/β-catenin axis promotes self-renewal and the differentiation of progenitor cells, while the Hippo pathway promotes kidney development. Hence, hypoplastic kidneys were noted in the case of YAP deletion (Hippo effector) [64,65]. Embryonic transcription factors (such as Oct4) and renal developmental genes (Pax2, Six2, Sall1, and Wt1) are typically expressed by ARPCs, which lack mature kidney cell markers.

## 5. Adult Renal Stem/Progenitor Cells

Tissue-specific adult stem cells have been identified in many organs, including the kidneys, bone marrow, gastrointestinal mucosa, prostate, liver, brain, and skin. The fact that postnatal renal tubules may be repaired after tubular necrosis indicates the presence of self-replicating cells in the adult kidney [66]. Research on chronic kidney disease (CKD) and the subsequent end-stage renal disease (ESRD) encouraged the isolation of adult stem cells and their potential role in tissue repair in the field of regenerative medicine to overcome dialysis and kidney transplantation [67,68,69]. Mesenchymal stem cells (MSC) have a crucial function during nephrogenesis. The arrest of the differentiation of embryonic progenitor cells following the nephrogenic lineage results in children’s Wilms tumor (WT), which has proved to be an effective biological system to study renal embryonic stem cells (ESC). In particular, WT cells shared high concordance with fetal kidneys in the expression of different markers (Pax2, Six1/2, NCAM, Fzd2, and Fzd7) [70]. However, the identification of embryonic stem cell markers is severely limited by the complete exhaustion of embryonic renal stem cells during nephrogenesis. Approximately 2% of the adult kidney’s total cells are remnant kidney ESCs, which are mostly found at the urinary pole of the Bowman’s capsule [71]. In turn, the adult kidney hosts two different pools of these cells: the resident adult renal stem/progenitor cells (ARPCs) and the circulating stem/progenitor cells. The latter group includes endothelial progenitor cells (EPCs), hematopoietic stem cells (HSCs), and bone marrow-derived MSCs (BMSCs) [72]. As mentioned above, progenitor cells have a more limited capability for differentiation than stem cells. Two different subpopulations of ARPCs were initially identified: the first in the tubule/interstitium and the second in the Bowman’s capsule. From the distal end of the proximal tubule, stem cells can migrate within this segment. In the Bowman’s capsule, ARPCs may acquire podocytes (PDX marker) and lose stem markers (CD133 and CD24) while moving from the urinary to the vascular pole. ARPCs in the Bowman’s capsule express CD106, unlike those in the tubules. CD133^+^CD24^+^CD106^+^ cells have a higher proliferation rate whereas CD133^+^CD24^+^CD106^−^ cells have a reduced self-renewal and differentiation capabilities. Therefore, CD106^−^ cells are thought to be in a more committed step toward differentiation [73]. Progenitor cells in the Bowman’s capsule also express kidney ESC and MSC (CD44) markers, as well as the stem transcription factors Oct-4 and Bmi-1. Apart from sharing CD133 and CD24 expression, these cells do not possess significant genomic differences [74,75]. ARPCs exhibit clonogenicity, stem cell markers and the ability to differentiate into other types of cells, including tubular epithelium-like, adipocyte-like, neuron-like, and osteogenic-like cells. Morphologically, they have less cytoplasm, fewer mitochondria, a mature brush border and no baso-lateral invaginations. Finally, CD133^+^ CD24^+^ cells are even thought to derive to renal ESCs because of their similar phenotype. ARPCs proliferate after acute and chronic tubular damage such as in transplanted patients undergoing delayed graft function [76]. They may express Toll-like receptor-2 (TLR2), which may be activated by various “damage-associated molecular pattern molecules” such as MCP-1 (monocyte chemotactic protein-1). MCP-1 expression is known to increase in the case of unilateral chronic ureteral obstruction [77,78]. Upon activation, TLR2 promotes ARPCs proliferation to induce the secretion of interleukins (IL-6 and IL-8) and MCP-1 (autocrine signaling loop) [79]. Over time, other stem/progenitor cells have been isolated. In the proximal tubules, Sox9^+^ Lgr4^+^ CD133^+^ cells may differentiate into proximal tubules, the loop of Henle, and distal tubules, but not into collecting ducts. They have brush border and epithelial polarity, but they lack Pax2 and MSC markers [80]. In the S3 segment of the nephron, Pax2^+^ cells have been found. Typically, they show an immature phenotype as well as progenitor and mesenchymal markers. Additionally, they could migrate into injured areas and in vivo differentiate into mature tubular epithelial cells but not into the vasculature [81]. Resident MSCs have been demonstrated to differentiate into mesodermal lineages, endothelial cells, and erythropoietin-producing fibroblasts when isolated from adult kidneys [82,83,84].

## 6. Renal Cancer Stem Cells

In recent years, there has been a growing interest in the identification of CSCs in renal cancer, their characterization, and comparison with the normal stem cell counterparts. Several markers have been studied in order to better identify RCC CSCs [85]. Prominin-1 (CD133) is a glycoprotein expressed on the cell membrane of stem and progenitor cells within normal tissues, and it has been proposed as a putative CSC marker across different tumor types. CD133^+^ RCC cells did not show in vivo tumorigenic capability, but when co-transplanted with RCC cells, they enhanced tumor engraftment, vascularization, and growth; however, different results have been obtained subsequently [86,87]. A wide variety of cells express CD24 on their surfaces, including hematopoietic cells, but it is typically expressed by progenitor and stem cells. When analyzing its role in RCCs, tumor grade, overall survival, and disease-free survival have been related to CD24 expression [88]. In a previous study, a subpopulation of CD133^+^CD24^+^ cells was isolated from ccRCC samples. Similar to their normal counterparts (ARPCs), these RCC-derived cells (RDCs) displayed self-renewal ability and clonogenic multipotency. Stemness-related elements (Nanog, Sox2, GATA4, and FoxA2) were confirmed while BMSC markers (CD90 and CD105) were not expressed. DNA microarray analysis was performed to better discriminate these RDCs from other cell types. It was observed that CTR2 (SLC31A2) characterized only RDCs so that neoplastic RDCs might be distinguished from normal ARPCs using CD133/CTR2 co-expression. In the presence of certain growth factors, RDCs might differentiate into osteocytes, adipocytes, or epithelial cells [89,90]. CTR2 regulates copper influx through cell membranes and its trafficking from cellular storage. However, drug accumulation and cytotoxicity may be affected by chaperones and transporters that regulate copper homeostasis. In particular, CTR2 may alter the accumulation of platinum-containing drugs via macropinocytosis and then promoting RDCs chemoresistance [91]. Xiao and colleagues further confirmed that CD133^+^CD24^+^ cells isolated from RCC cells express stemness-related genes and assessed the Notch signaling pathway. Self-renewal potential, resistance to cisplatin and sorafenib, in vivo tumorigenicity, and invasion and migratory capability were typically recognized in these CD133^+^CD24^+^ cells. Aberrant Notch pathway activation resulted in the upregulation of genes related to drug resistance (MDR1), self-renewal (Oct4 and Klf4), and anti-apoptotic activity (Bcl-2). These properties were partially lost upon blocking Notch pathways via exogenous (MRK-003) or endogenous (Numb) inhibitors since gene expression was reduced [92].

CD105 (endoglin) is a transmembrane glycoprotein that forms part of the transforming growth factor-β (TGFβ) complex. Its activation promotes Smad proteins, thus regulating various processes such as proliferation, migration, differentiation, and angiogenesis. Endoglin is typically expressed on endothelial cells where it is activated by TGFβ and hypoxia and silenced by tumor necrosis factor α (TNFα) [93]. A subpopulation of CD105^+^ cells from RCC was shown to express mesenchymal markers (CD44, CD90, CD29, CD73, CD146, and vimentin), embryonic stem cell markers (Oct3/4, Nanog, Musashi, and Nestin), and the embryonic renal marker Pax2, but they lacked differentiative epithelial markers (i.e., cytokeratin—CK). Epithelial, endothelial, and CD105^−^ cells may arise from CD105+ CSCs differentiation. In SCID mice, a modest number of cells were able to produce serially transplantable carcinomas (the same histological pattern for the origin of tumor) with a large proportion of differentiated CD105^−^ cells and a small fraction of CD105^+^ population [94]. In turn, CD105^+^, CD44^+^, as well as CD105^−^, CD44^−^, and CD105^−^/CD44^−^ cells were able to give rise to tumors when injected into mice [95]. Additionally, CD105^+^ CSCs are able to secrete exosomes and microvesicles containing mRNAs (VEGF, FGF, MMP2 and 9) promoting angiogenesis and metastatic niche formation as well as the impairment of T cell activation and dendritic cell activation [96,97,98].

RNA alternative splicing give rise to different isoforms of CD44, which are then involved in diverse biological processes, such as cell–cell interaction, cell adhesion, proliferation, migration, differentiation, and angiogenesis. Glycosaminoglycan hyaluronan (HA) represents the main ligand of this transmembrane glycoprotein, whereas other extracellular matrix (ECM) components may interact with CD44 (i.e., collagen, growth factors, and metalloproteinases). Its binding promotes multiple signaling pathways such as TGFβ, MAPK, PI3K/AKT and receptors tyrosine kinases (RTKs), thus encouraging cell proliferation, survival, invasion, and CSCs homing in different tumors [99,100]. Wnt/β-catenin and protein kinase C (PKC) pathways may be modulated by CD44 [101]. CD44 has been stated to modulate CSC niche owing to its interaction with ECM elements. Since CD44 expression was related to Fuhrman grade, primary tumor stage, histological subtypes, and poor patient prognosis, it may represent a potential marker for CSCs in RCC [102,103].

CXC chemokine stromal cell-derived factor 1 (SDF1 or CXCL12) selectively binds to the CXC-chemokine receptor 4 (CXCR4 or CD184) [104,105,106]. Downstream effectors include PLC/MAPK, PI3K/AKT, JAK/STAT, and the Ras/Raf pathways. Several biological processes are activated, such as proliferation, survival, migration, stemness, and angiogenesis. In renal and other solid tumors, CXCR4^+^ cells migrate towards tissues expressing high levels of SDF1 to metastasize. CXCR4^+^ cells from RCC cell lines have already shown the high expression of stem cell-associated genes (Oct4, Sox2, and Nanog) as well as resistance to therapy (TKI). Tumor growth was impaired by blocking CXCR4 with ADM3100 or small interfering RNA (siRNA). Hypoxia and the loss of pVHL were observed to increase CXCR4 and MMPs expression in recent studies. CD133^+^CXCR4^+^ cells were noted to locate in perinecrotic areas of RCC where they expressed HIF1α [107]. In addition, hypoxia promoted the tumorigenicity of CD133^+^CXCR4^+^ cells and HIF2α promoted the expansion of CXCR4^+^ CSCs [108,109]. Marginal CXCR4/CD105 co-expression was confirmed; therefore, CD105^+^ cells may even represent a major CXCR4 subpopulation [110]. Perhaps in association with another marker, CXCR4 might be investigated as a possible CSC marker in RCC. Fendler et al. [111] performed the transcriptional profiling of CXCR4/MET/CD44^+^ cells isolated from ccRCCs specimens. These authors showed that a greater number of CXCR4/MET/CD44^+^ cells was associated with higher pathological stage and Fuhrman grade, with venous and lymphatic invasion, and distant metastases. The analysis of gene and protein expression demonstrated that Wnt and Notch signaling was activated, and that their inhibition blocked these CSCs. Beta-catenin and Jade1 are stabilized so as Notch signaling is activated owing to pVHL loss [111]. Notch activation in RCC CSCs promotes CXCR4 upregulation then encouraging SDF-1-induced chemotaxis [92].

Acetaldehyde dehydrogenase 1 (ALDH1) takes part in alcohol metabolism in the hepatocyte cytoplasm, and is known to be crucial for cellular differentiation, proliferation, motility, embryonic development, and organ homeostasis [112]. Indeed, in healthy human stem cells, ALDH1 may also convert the retinal to retinoic acid (RA). Upon activating retinoic acid receptor (RAR), retinoic acid X receptor (RXR), and nuclear hormone receptor peroxisome proliferator activated receptor β/δ (PPAR β/δ), RA will modulate the expression of several genes. In cancer, metabolism reprogramming, DNA repair and stem-like features depend on different pathways linked to ALDH1 (RA, ROS, USP28/MYC, HIFα/VEGF, Wnt/β-catenin). Its prognostic significance in RCC remains unclear, although it has been regarded a reliable marker of CSCs in several solid cancers. For instance, it may recruit myeloid-derived suppressor cells (MDSCs) in the TME of breast cancer, thus limiting cancer immunity. Chemosensitivity is increased when inhibiting its enzymatic activity [113,114].

DnaJ homolog, subfamily B, member 8 (DNAJB8) is a member of HSP40 family of the heat shock proteins. It is typically expressed in postmeiotic sperm and spermatid and is suggested to regulate androgen signaling during spermatogenesis. Chaperones prevent cytotoxic stress by controlling protein folding. DNAJB8 might have oncogenic potential since it strongly suppresses misfolded protein aggregation. This HSP plays a role in the maintenance of RCC CSCs as its targeting fully blocked tumor formation in mice, suggesting that it may be a target for immunotherapy [115,116] (Table 1).

MicroRNAs (miRNAs) regulates gene expression and their roles in CSCs have been elucidated for some cancers. In RCC, increased sphere formation was shown after the inhibition of miR17 [117].

The Hoechst exclusion assay, which was first introduced in 1996, is another functional technique to identify CSCs by using a family of blue dyes called “Hoechst stains” (bis-benzimides used to stain DNA). Stem cells have a high efflux capacity, which allows them to remove Hoechst dye from the intracellular space and appear as a side population (SP). Using cell separation techniques, stem cell markers and the Hoechst exclusion assay can both be combined to enhance CSC in a biological sample. However, Hoechst staining may be excluded by some differentiated tumor cells that express high levels of ABCG2 and ABCB1 [118].

Hypoxia is thought to play a central role in the maintenance of normal embryonic and adult stem cells. Low oxygen pressure in the niche may reduce ROS-associated genotoxic oxidative damage, therefore promoting self-renewal and inhibiting differentiation. Because of mutations in VHL, which are carried by most of ccRCC, the constitutive activation of HIFs defines a pseudo-hypoxic phenotype. Transcriptomic analysis in RCC CSCs lead to the sequencing of different long non-coding RNA (lncRNAs). Hypoxia has been demonstrated to reduce androgen receptor (AR) in RCC, which may, in turn, regulate lncTCFL5-2 expression. In particular, lncTCFL5-2 seems to be enhanced by knocking down AR. The lncTCFL5-2/YBX1 complex may translocate to the nucleus where target genes are promoted, such as Sox2, CD133, and CD24 [119].

Single-cell RNA-seq analysis was performed in collecting duct renal cell carcinoma (CDRCC). EZH2 was shown to be significantly overexpressed in the CSC subpopulation to control its gene expression and self-renewal property. From this study, PARP, PIGF, HDAC, and FGFR inhibitors emerged as potential candidates for targeting CSCs [120].

Zhou et al. [29] clustered ccRCCs specimens into three subgroups based on stem/progenitor signatures. Significant antitumor immune infiltration (M1 macrophages, activated dendritic cells and CD4/CD8 T cells), enhanced HLA-I molecule expression, and cytolytic activity was associated with increased stemness signature. In contrast, high-stemness subgroup showed increased immune checkpoint molecules, cancer-associated fibroblasts (CAFs), MDSCs, and regulatory T cells (Tregs) with robust immunosuppressive properties. In this scenario, these authors even hypothesized that a stemness-related gene signature may be useful to predict anti-PD-1 responses [29]. Over time, different agents have been assessed to target RCC CSCs. In response to IL-15 (a regulator of kidney homeostasis), CD105^+^ CSCs lost their capacity to initiate tumors, to express stem cell markers, and to form spheres. However, they also gained polarity, transmembrane resistance, epithelial markers, vinblastine, and paclitaxel sensitivity [121]. PI3K/Akt/mTOR axis has already been established to play an essential role in CSC biology and mTOR inhibitors have been proved to eradicate CSCs in different human cancers (neuroblastoma, nasopharyngeal, colon, and pancreatic cancers). Further studies are needed to confirm whereas combination therapies using mTOR inhibitors are indeed effective in targeting both renal cancer cells and CSCs [122,123]. Bone morphogenetic protein-2 (BMP-2) encodes a member of the TGF superfamily and is known to regulate different cellular processes such as cell differentiation, proliferation, morphogenesis, survival, and apoptosis. Depending on the cancer type, BMP-2 has been shown to either drive or prevent tumor growth. BMP-2 inhibits the tumor-initiating ability of renal CSCs and promotes bone formation in vivo. In particular, BMP-2 reduces the expression of embryonic stem cell markers and renal markers in CSCs (Oct3/4A, Nanog, and Pax-2), and increased the expression of osteogenic markers (Runx2 and collagen type I) [124]. Low molecular weight inhibitors fumitremorgin C and tryprostatin, as well as monoclonal antibodies, cyclosporin A, VX710, or tariquidar, have been attempted to eradicate CSCs exploiting ABC transporters [125]. It has also been suggested to use monoclonal antibodies or inhibitors against their surface markers. CD133 has been used as a target for the treatment of glioblastoma, lung cancer, and liver cancer. Many studies have shown that salinomycin is able to kill CSCs in a variety of human cancers, including gastric cancer, lung adenocarcinoma, osteosarcoma, colorectal cancer, squamous cell carcinoma, and prostate cancer. This result was most likely achieved by interfering with ABC transporters, Wnt/β-catenin signaling, or additional CSC pathways [126]. Nanoparticles have been introduced to target CSCs since they are carriers for chemotherapeutic or nucleic acid drugs that accumulate at tumor sites. Combinations of paclitaxel/salinomycin-loaded PEG-b-PCL polymeric micelles have been designed for breast cancer treatment. Recent studies showed that salinomycin targeted CSCs whereas paclitaxel targeted most cancer cells, producing a higher antitumoral action in vitro and in vivo than either agent alone [127,128]. This combination therapy may represent an effective strategy to improve the treatment of solid tumors as it acts in the eradication of both cancer cells and their stem counterpart.

## 7. Conclusions

**Figure 2:**
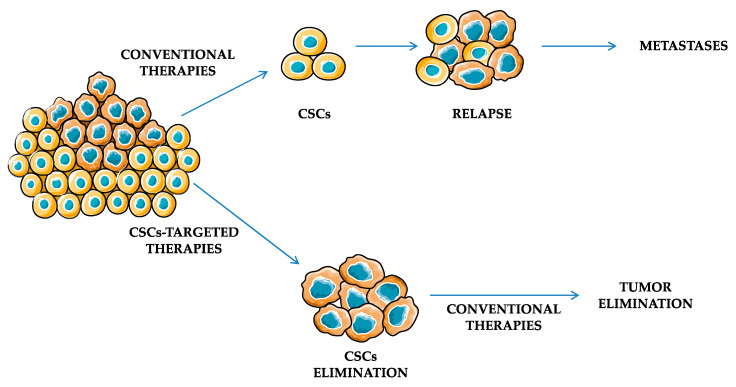
CSCs appear to be resistant to conventional therapies, thus promoting relapses and metastases. Development of CSCs-targeted agents may facilitate tumor elimination.

Most of the available cancer treatment strategies target somatic tumor cells rather than CSCs, which are assumed to be responsible for tumor recurrence and metastasis (Figure 2).

The lack of effective putative markers is the consequence of the conflicting results so far reported in the literature. In addition to not being specific between tumor types, it has been postulated that some biomarkers may be transient as they may become obsolete at particular stages of tumorigenesis. Optimizing renal CSC isolation and characterization techniques will be crucial for the development of effective therapies against CSCs. The development of agents targeting CSC-signaling specific pathways and not only surface proteins may ultimately become of utmost importance for patients with RCC.

## Figures and Tables

**Table 1:** Putative markers of renal CSCs.

Marker	Properties	Reference
CD133/CD24	Clonogenic, chemoresistance	[89,90]
CD105	Sphere formation, clonogenic, differentiation, tumorigenicity	[93]
CD44	Niche homing, patients’ prognosis	[101,102]
CXCR4	Sphere formation, tumorigenicity, chemoresistance	[109,110]
ALDH1	Tumorigenicity, chemoresistance	[112]
DNAJB8	Tumorigenicity	[115]

## Data Availability

No new data were created.
